# Therapeutic potential of *Garcinia kola* against experimental toxoplasmosis in rats

**DOI:** 10.1093/braincomms/fcae255

**Published:** 2024-08-06

**Authors:** Nene Ahidjo, Frederic Maidawa Yaya, Wepnyu Y Njamnshi, Judith C Rissia-Ngo Pambe, Ethel W Ndianteng, Caroline N C Nwasike, Christelle Kemmo, Arnaud C Choupo, Luc Yvan Meka’a Zang, Anatole C Pieme, Lorella Vecchio, Bonaventure T Ngadjui, Alfred K Njamnshi, Paul F Seke Etet

**Affiliations:** Brain Research Africa Initiative (BRAIN), Yaoundé, Cameroon; Faculty of Medicine and Biomedical Sciences, Neuroscience Laboratory, The University of Yaoundé I, Yaoundé, Cameroon; Faculty of Medicine and Biomedical Sciences, Neuroscience Laboratory, The University of Yaoundé I, Yaoundé, Cameroon; Department of Physiological Sciences and Biochemistry, Faculty of Medicine and Biomedical Sciences, Center for Sustainable Health and Development, University of Garoua, Garoua, Cameroon; Brain Research Africa Initiative (BRAIN), Yaoundé, Cameroon; Faculty of Medicine and Biomedical Sciences, Neuroscience Laboratory, The University of Yaoundé I, Yaoundé, Cameroon; Department of Morphological Sciences and Pathological Anatomy, Faculty of Medicine and Biomedical Sciences, University of Garoua, Garoua, Cameroon; Faculty of Medicine and Biomedical Sciences, Neuroscience Laboratory, The University of Yaoundé I, Yaoundé, Cameroon; Faculty of Medicine and Biomedical Sciences, Neuroscience Laboratory, The University of Yaoundé I, Yaoundé, Cameroon; Faculty of Medicine and Biomedical Sciences, Neuroscience Laboratory, The University of Yaoundé I, Yaoundé, Cameroon; Faculty of Medicine and Biomedical Sciences, Laboratory of Biochemistry, University of Yaoundé I, Yaoundé, Cameroon; Faculty of Medicine and Biomedical Sciences, Neuroscience Laboratory, The University of Yaoundé I, Yaoundé, Cameroon; Faculty of Medicine and Biomedical Sciences, Laboratory of Biochemistry, University of Yaoundé I, Yaoundé, Cameroon; Brain Research Africa Initiative (BRAIN), Yaoundé, Cameroon; Department of Physiological Sciences and Biochemistry, Faculty of Medicine and Biomedical Sciences, Center for Sustainable Health and Development, University of Garoua, Garoua, Cameroon; Department of Organic Chemistry, The University of Yaoundé I, Yaoundé, Cameroon; Brain Research Africa Initiative (BRAIN), Yaoundé, Cameroon; Faculty of Medicine and Biomedical Sciences, Neuroscience Laboratory, The University of Yaoundé I, Yaoundé, Cameroon; Brain Research Africa Initiative (BRAIN), Yaoundé, Cameroon; Faculty of Medicine and Biomedical Sciences, Neuroscience Laboratory, The University of Yaoundé I, Yaoundé, Cameroon; Department of Physiological Sciences and Biochemistry, Faculty of Medicine and Biomedical Sciences, Center for Sustainable Health and Development, University of Garoua, Garoua, Cameroon

**Keywords:** *Toxoplasma gondii*, low-protein diet, *Garcinia kola*, neuronal loss, neuroprotection

## Abstract

Cerebral toxoplasmosis, the most common opportunistic infection in immunocompromised individuals, is increasingly reported in immunocompetent individuals due to mutant strains of *Toxoplasma gondii*, which, furthermore, are reported to be resistant to available treatments. We assessed the therapeutic potential of *Garcinia kola*, a medicinal plant reported to have antiplasmodial and neuroprotective properties, against experimental toxoplasmosis in rats. Severe toxoplasmosis was induced in male Wistar rats (156.7 ± 4.1 g) by injecting them with 10 million tachyzoites in suspension in 500 µl of saline (intraperitoneal), and exclusive feeding with a low-protein diet [7% protein (weight by weight)]. Then, animals were treated with hexane, dichloromethane, and ethyl acetate fractions of *Garcinia kola*. Footprints were analysed and open-field and elevated plus maze ethological tests were performed when symptoms of severe disease were observed in the infected controls. After sacrifice, blood samples were processed for Giemsa staining, organs were processed for haematoxylin and eosin staining, and brains were processed for Nissl staining and cell counting. Compared with non-infected animals, the infected control animals had significantly lower body weights (30.27%↓, *P* = 0.001), higher body temperatures (*P* = 0.033) during the sacrifice, together with signs of cognitive impairment and neurologic deficits such as lower open-field arena centre entries (*P* < 0.001), elevated plus maze open-arm time (*P* = 0.029) and decreased stride lengths and step widths (*P* < 0.001), as well as neuronal loss in various brain areas. The ethyl acetate fraction of *Garcinia kola* prevented or mitigated most of these signs. Our data suggest that the ethyl acetate fraction of *Garcinia kola* has therapeutic potential against cerebral toxoplasmosis.

## Teaser

Ahidjo *et al*. report that treatment with fractions of *Garcinia kola*, in particular, the ethyl acetate fraction, mitigated or prevented the neurotoxoplasmosis-like cognitive and motor signs typically observed in rats following exclusive feeding with a low-protein diet. Hence, fractions of *G. kola* may have a therapeutic potential against cerebral toxoplasmosis.

## Graphical Abstract

**Graphical Abstract:**
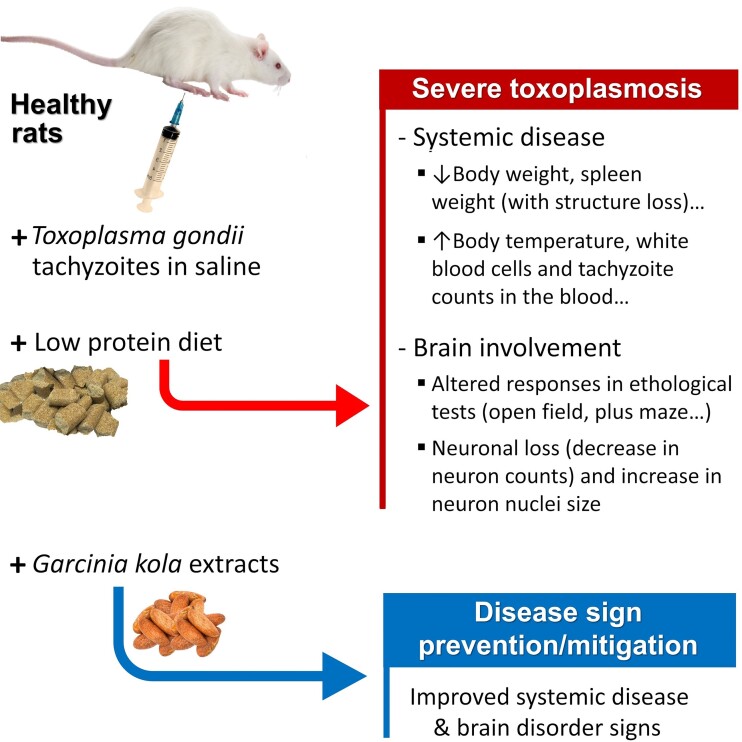

## Introduction

The apicomplexan parasite *Toxoplasma gondii* poses a serious public health problem in low- and middle-income countries (LMICs) partly due to the heavy burden of HIV/AIDS cases and poverty-related healthcare challenges that highly affect pregnant women and infants.^1,2^ Traditionally, *T. gondii* is known to be problematic in immunocompromised humans, and infections are mostly latent and seem asymptomatic in immunocompetent individuals; thus, the parasite has infected one-third of the worlds’ population without raising alarms.^3,4^ Alarmingly, a growing number of studies have reported links between *T. gondii* infection and the development of various neuropsychiatric disorders in immunocompetent individuals and cases of toxoplasmosis in immunocompetent individuals are increasingly reported.^5-7^  *Toxoplasma gondii* has developed various strategies to adapt to its host, including the ability to evade host immune responses, including by hiding in host brain starting from early infection stages; and to switch between its replicative (tachyzoite) and latent drug-resistant tissue cyst (bradyzoites).^8,9^ The persistence of tachyzoites in neurons and cysts in brains of immunocompetent hosts results in brain tissue responses to parasite antigens, which were linked to behavioural changes in rodents,^10-12^ and more recently, to the risk for developing neuropsychiatric disorders^3,13,14^ and brain tumours in humans.^15,16^

Cases of severe toxoplasmosis are increasingly reported in immunocompetent individuals partly due to mutant strains of *T. gondii* which, also associate with increased clinical severity in immunocompromised patients.^17,18^ These mutant strains are also resistant to most of the current drugs, including the widely used anti-*Toxoplasma* drugs such as sulphadiazine and pyrimethamine.^19,20^ In addition, environmental factors affecting immune system functions such as diet low in proteins, a common problem in LMICs, also contribute to the development of severe toxoplasmosis in immunocompetent individuals.^21,22^ Thus, new therapeutics that have a good central nervous system penetration, and possibly also effective in undernourished infected cases, are direly needed against severe forms of toxoplasmosis. Acute experimental toxoplasmosis in animals is being used as models for detecting agents with promising potential against severe forms of toxoplasmosis.^23,24^

We assessed the therapeutic potential of extracts of seeds of *Garcinia kola*, which were reported to possess antiparasitic, neuroprotective, antioxidative and anti-inflammatory activities,^25-29^ in a rat model of severe toxoplasmosis with both systemic and cerebral components, namely Wistar rats infected with a clinically relevant cystogenic *T. gondii* strain and exclusively fed with a low-protein diet (LPD).

## Materials and methods

### Animals

Two-month-old male (156.7 ± 4.1 g, *N* = 30) Wistar rats were obtained from the Faculty of Science of the University of Yaoundé I (Yaoundé, Cameroon) and acclimatized to Neuroscience Laboratory conditions (Faculty of Medicine and Biomedical Sciences, University of Yaoundé I). Animals were housed in groups of three, had free access to water and food, and were kept at 25°C under a 12:12 light–dark cycle. Experimental procedures were approved by the Institutional Ethics Committee (No. 0634/UY1/FMSB/VDRC/DAASR/CSD). The animals were handled considering ethical rules relating to the protection of animals used for scientific purposes, in particular, the European Commission Directive (2010/63/EU).

### Experimental procedures

Thirty male rats were randomly divided in five groups (*N* = 6/group), of which a non-infected control group was injected with sterile saline [intraperitoneal (i.p.)] and fed with standard rat chow [15% protein, 3% fat and 7% simple sugars (w/w)] and four groups were infected with *T. gondii* tachyzoites and fed with a LPD [normal chow-like diet with 7% protein (w/w)].^30,31^ The infected groups encompassed an untreated group injected with sterile saline (i.p.) (infected control group) and three groups treated once daily with hexane, dichloromethane (DCM) and ethyl acetate (EA) fractions of the methanolic extract of *G. kola* at doses equivalent to the content of the dose 100 mg/kg of the crude extract, which was reported to have a strong neuroprotective activity and to be safe.^26,27,32^ The animals were infected with *T. gondii* tachyzoites (TS-4/ATCC 40050) that were kindly provided by the Antimicrobial & Biocontrol Agents Unit of the University of Yaoundé I, by single injection of 10 million tachyzoites in suspension in 500 µl of sterile saline (i.p.), considering pilot studies in our laboratory and previous reports.^33,34^ The infection was confirmed when tachyzoites were observed in blood smears collected from the tail vein 3 days after inoculation, both inside and outside leucocytes.^33^

Then, after confirming the success of the infection, the animals were continuously monitored, video-recorded and their body weight and body temperature (inner ear and infrared thermometry) were measured every 3 days. Treatment with fractions of *G. kola* started 7 days post-infection (dpi), around the period where significant decreases in body weight occurred in infected animals. After 10 days of treatment, as symptoms of severe disease were observed in the infected control animals, footprints were analysed and two ethological tests were performed sequentially, namely the open-field test (OFT) and the elevated plus maze (EPM) paradigm. The day after (dpi 17), all animals were sacrificed under deep anaesthesia to avoid prolonging the animal suffering unnecessarily. Organs and blood samples were collected. For histopathological analysis, blood samples were processed for Giemsa staining and organs for haematoxylin and eosin (H&E) staining. In addition, phytochemical screening of the fractions of *G. kola* was performed.

### Plant material processing and phytochemical screening

#### Seed processing

*Garcinia kola* seeds were harvested during the maturing period (August) in Bamenda, North West region of Cameroon. Seeds were authenticated in the National Herbarium of Cameroon (R. Louzey number 11.981, code number 28837/HNC) and a sample was stored for future references. Then, seed coats were peeled off and seeds were dried under the shade. Dried seeds were ground into powder, and the powder was extracted with methanol using a Soxhlet extractor (65°C, 5 h). Methanol was evaporated using a rotary evaporator, and the methanolic extract obtained was fractionated by sequential exposure to hexane, DCM and EA solvents using a Soxhlet extractor to extract the fractions, and a rotary evaporator was used for the removal of excess solvent as described previously.^26^

#### Qualitative phytochemical screening

We screened for alkaloids, phenols, flavonoids, terpenoids, tannins and saponins using standard procedures. Briefly, in a test-tube containing 1 ml of extract solution (2% in 1:1 ethanol–distilled water) following procedures were performed to detect: (i) polyphenols, five drops of iron(III) chloride (FeCl_3_) were added, followed by three drops of potassium cyanide (positive result if the colour in the test-tube turns greenish); (ii) terpenes, 1 ml of glacial acetic acid and 3–5 drops of concentrated sulphuric acid (H_2_SO_4_) (positive result if the colour in the test-tube turns brownish red); (iii) alkaloids, 3–5 drops of Valse Mayer’s reagent, i.e. 1.3 g of mercury chloride (HgCl_2_) and 5 g of potassium iodide for a volume of 100 ml (positive result if yellowish white precipitate or creamy white precipitate appears); (iv) flavonoids, 1 ml of sodium hydroxide (NaOH) 2 N was added, followed by 2–3 drops of concentrated H_2_SO_4_ (positive result if the colour in the test-tube turns orange-yellow); (v) quinones, few drops of 10% NaOH (positive result if the colour in the test-tube turns red); (vi) steroids, 2 ml of acetic anhydride (C_4_H_6_O_3_) and 2 ml of concentrated H_2_SO_4_ (positive result if the colour in the test-tube turns violet to blue or green); (vii) coumarins, 1 ml of distilled water and few drops of 10% FeCl_3_ and nitric acid (HNO_3_) (positive result if the colour in the test-tube turns green or blue to yellow); (viii) anthocyanins, 1 ml concentrated H_2_SO_4_ and 1 ml of diluted ammonia (NH_3_) (positive result if the colour increases in the acidic medium then turns to purplish blue in the basic medium); (ix) mucilage, 3 ml of absolute ethanol (positive result if fluffy precipitate appears upon agitation); (x) saponins, 5 ml of distilled water (positive result if presence of a stable foam is observed after 15 min); (xi) tannins, 0.5 ml of STIANYS reagent and heat it in a water bath at 90 °C for 15 min (presence of catechic tannins if soluble red precipitate appears), then filtration and saturation of the filtrate with pulverized sodium acetate (C_2_H_3_NaO_2_) followed by 1% FeCl_3_ (presence of gallic tannins if dark blue precipitate appears).

#### Quantitative phytochemical screening

Quantitative phytochemical screening was performed for the secondary metabolites of *G. kola*, namely polyphenolic compounds, flavonoids, flavanols and tannins that reported to possess neuroprotective, antioxidants, anti-inflammatory and anti-parasitic activities.^25-29^ Methods based on standard spectrophotometry was used, namely: (i) Folin–Ciocalteu method to estimate the amount of total phenolic content, based on the ability of polyphenols to reduce the phosphotungstic and phosphomolybdic acids of the Folin–Ciocalteu’s reagent in an alkaline medium into a mixture of blue tungsten and molybdenum oxides, which absorb light at 765 nm^35^; (ii) the method described by Zhishen *et al.*^36^ to estimate the total flavonoid content, based on the ability of aluminium chloride (AlCl_3_) to form acid-stable complexes with the C-4 keto group and either the C-3 or C-5 hydroxyl group of flavones and flavanols, respectively, and to also form acid labile complexes with the orthodihydroxyl groups in the A- or B-rings of flavonoids that result in pink colour formation that can be measured at 510 nm; (iii) the method described by Kumaran and Karunakaran^37^ to estimate the flavanol content, based on the ability of AlCl_3_ to chelate flavanols in an ethanolic solution, with colorimetric changes measurable between 380 and 400 nm and (iv) the method described by Lau *et al.*^38^ to estimate the tannin content of a phytoextract, based on the ability of tannins to reduce Fe^3+^ to Fe^2+^, which reacts in turn with 1,10-phenanthroline at pH 4.4 to form a coloured complex that can be measured at 540 nm.

### Footprint analysis and behavioural tests

#### Footprint analysis

The footprint analysis was performed for the assessment of motor abilities, in particular balance and gait.^26,39^ Rat paws inked and the animals were allowed to walk freely along an enclosed box (50-cm long, 10-cm wide and 20-cm high walls) with a clean sheet of paper placed on the floor. For each animal, a valid trial over three consecutive trials was considered, in order to avoid abnormal patterns associated with the habituation phase.^40^ The footprint patterns and contacts were digitized using a high-resolution scanner (HP Scanjet Pro 3000 s3, Hewlett Packard, Palo Alto, CA, USA) and analysed. The step width and length were determined using MATLAB Image Processing Toolbox (MathWorks, Natick, MA, USA).

#### Open-field test

The open-field arena was a 50-cm high wooden box with 100 cm × 100 cm floor including a 40 cm × 40 cm central zone and a peripheral zone. In this test, a rat was placed facing the wall in a corner of the arena and the animal's activity was video-recorded for 10 min. Vertical and horizontal activities were simultaneously video-recorded using a computerized camera (LifeCam Studio, Microsoft, Redmond, Washington, USA) and placed 130 cm above the arena with a 45° angle. The floor and walls of the arena were cleaned with 70% alcohol solution after each trial, to prevent bias due to olfactory cues. The distance travelled in the arena, as well as the number of entries and time spent in the arena zones were determined using motion tracking on image sequences in the Image Processing Toolbox of MATLAB software (MathWorks, Natick, MA). The characteristics of episodes of stretch–attend posture (SAP) (hind paws being stationary while the body is stretched forward for more than 3 s), rearing (on hind paws and against the wall), paw licking (grooming less than 5 s) and grooming (episodes of atleast 5 s) were scored from video-recordings offline.

#### EPM paradigm

The EPM apparatus was raised 50 cm above the ground level and consisted of two open arms (50 cm × 10 cm, no wall), two arms (50 cm × 10 cm) enclosed by wooden walls (40 cm high) and a common central platform (10 cm × 10 cm). Each rat was placed on the central platform facing an open arm and behaviour was recorded for 5 min. The performance on the EPM was recorded using a computerized video-recording system including a camera (LifeCam Studio) placed 150 cm above the centre of the apparatus. After each trial, the floor and walls of the EPM were cleaned with a 70% ethanol solution. Video-recordings were scored offline for the number of entries, time spent and distance travelled in the arms, as well as episodes of head dipping above the open-arm edge, grooming and rearing. An entry occurred when all four limbs were within an arm.

### Histopathological analyses

#### Processing of blood samples

A thin blood smear was obtained by placing a drop of blood onto an end of a clean microscopic slide and by spreading it gently using the edge of a second slide. Then, blood smears were processed for May-Grünwald Giemsa staining using standard procedures: the blood smear mounted on a slide was covered with 1 ml of the May-Grünwald solution for 3 min, 1 ml of the buffer solution was carefully added and the mixture was left in contact for 1 min. Then, the excess dye was discarded by draining or rapid rinsing. The smear was covered with Giemsa R stain diluted 1/30 in a buffer (10 min) and the slide was quickly rinsed with running water for 10 s. Then, the stained smear was dehydrated and mounted using DPX medium (reference 4458, Sigma-Aldrich, Burlington, MA, USA). Observations of slides were made at ×10, ×40 and ×100 objectives and pictures were taken at ×40 using a digital microscope camera (MT series 9 MB USB camera, AmScope, Irvine, CA, USA).

Counting frames allowing rigorous counting were delimited on micrographs, and white blood cells (WBCs), *T. gondii* tachyzoites and cysts present in the blood were counted using Cell Counter macro in the ImageJ software version 1.54 g (National Institutes of Health, Bethesda, MA, USA). Parasites present in parasitized blood cells were not counted. To quantify the circulating parasites, the parasites were tallied against WBCs until 1000 WBCs were counted. The results were expressed as parasites per microliter of blood, assuming 8000 WBCs per microliter of blood.


$$\mathrm{Tachyzoites}\;\mathrm{or}\;\mathrm{cysts}\;\mathrm{per}\;\mathrm{microliter}\;\mathrm{of}\;\mathrm{blood}=\left(\frac{\mathrm{tachyzoites}\;\mathrm{or}\;\mathrm{cysts}}{\mathrm{WBCs}}\right)\;\times \;8000.$$

For each animal, all WBCs were tallied against the estimated number of red blood cells (RBCs) until 1 000 000 (determined using Nucleus Counting macro of Image J) and counted in at least three randomly selected micrographs, assuming 5 000 000 RBCs per microliter of blood.


$$\mathrm{WBCs}\;\mathrm{per}\;\mathrm{microliter}\;\mathrm{of}\;\mathrm{blood}=\left(\frac{\mathrm{WBCs}}{\mathrm{RBCs}}\right)\times 5\;000\;000.$$

#### Tissue processing and H&E staining

After harvesting, the organs (kidney, liver, spleen and brain) were immersed in 10% formaldehyde for 8 h. Then, they were processed for inclusion in paraffin. Blocks were cut entirely in organ transversal plane (thickness: 5 µm), and sections were mounted on slides. Afterwards, for each organ, a series of sections was randomly selected and stained with H&E using standard protocols. Briefly, the sections were deparaffinized with xylene (1-min immersion), rehydrated with decreasing concentrations of ethanol (diluted with water distilled: 100, 96, 70, 50%, distilled water). After 10 min in haematoxylin, the slides were washed abundantly in running water, then placed for a few moments in phosphate buffer until section bluing. Then, the sections were rinsed with distilled water, placed for 5 min in eosin, washed briefly in running water, dehydrated in increasing concentrations of ethanol, put in xylene (×2, 5 min), and mounted between slide and coverslip using DPX. The slides were examined with a microscope at ×20, × 40 and × 100 objectives, and pictures were taken at objective × 40 with a digital microscope camera (MT series USB camera).

#### Nissl staining and neuron counting on brain sections

Three sections throughout the brain anterior zone including the anterior cingulate cortex, the brain posterior zone including the posterior parietal cortex and the cerebellar zone including the dentate nucleus were selected using systematic random sampling, i.e. starting from a random point, then progressing with a fixed periodic interval (8 µm in this study). The selected sections were processed for cresyl violet (Nissl) staining using standard procedures. Briefly, after deparaffinizing with xylene ( × 2, 10 min), sections were hydrated by immersion in ethanol baths diluted with distilled water of decreasing concentration (100, 100, 96, 70 and 50%, 5 min for each step), stained by immersion in cresyl violet solution for 5 min (for 100 ml: 0.02 g of cresyl violet acetate and 0.25 ml of glacial acetic acid in distilled water), rinsed in three changes of distilled water, dehydrated by immersion in ethanol baths of increasing concentration (50, 70, 96, 100 and 100%), cleared in three changes of xylene, and mounted with DPX. Then, neuron nuclei were counted in: (i) the anterior cingulate cortex that is involved in action, emotion and memory, with a cardinal role in controlling the expression of contextual fear generalization^41,42^; (ii) the medial septal nucleus that is critical for learning and memory and which prevented sepsis-induced cognitive deficits in mice^43,44^; (iii) the posterior parietal cortex, which is an associative region comprising the primary somatosensory areas^45^; (iv) the perifornical zone of the lateral hypothalamic area, which is critical for various physiological functions, including the promotion and stabilization of active-arousal and drive to eating^46,47^; (v) the cerebellar molecular layer which contains inter-neurons that are key elements of cerebellar network computation and behaviour^48^ and (vi) the cerebellar dentate nucleus whose neuron loss accounts for cerebellar symptoms in various neurodegenerative disorders.^49,50^ Neuron nuclei counts were performed on micrographs (taken at × 40 objective) of the areas of interest using semi-automatic counting with ImageJ software. More specifically, neuron nuclei were counted automatically using Nucleus Counting macro of Image J, and afterwards, an observer manually checked the counted particles to ensure that they all were neuron nuclei, and that nuclei crossing the lower and left borders were discarded. ImageJ software also automatically determined the average size of the nuclei counted and the percent of total area occupied by these nuclei.

### Data analysis

Statistical significance of inter-group differences in body weight, body temperature, and cognitive and motor indicators revealed by footprint analysis, the EPM, and the OFT, as well as neuron nuclei counts and area were assessed using one-way ANOVA, followed by least square difference (LSD) test for inter-couple comparisons (OriginPro 8 software, OriginLab Co., Northampton, MA, USA). Differences with *P* < 0.05 were considered significant. Data were presented as mean ± SEM.

## Results

### Physiological parameters and gait

#### Body weight, temperature and blood glucose level

**Figure 1:**
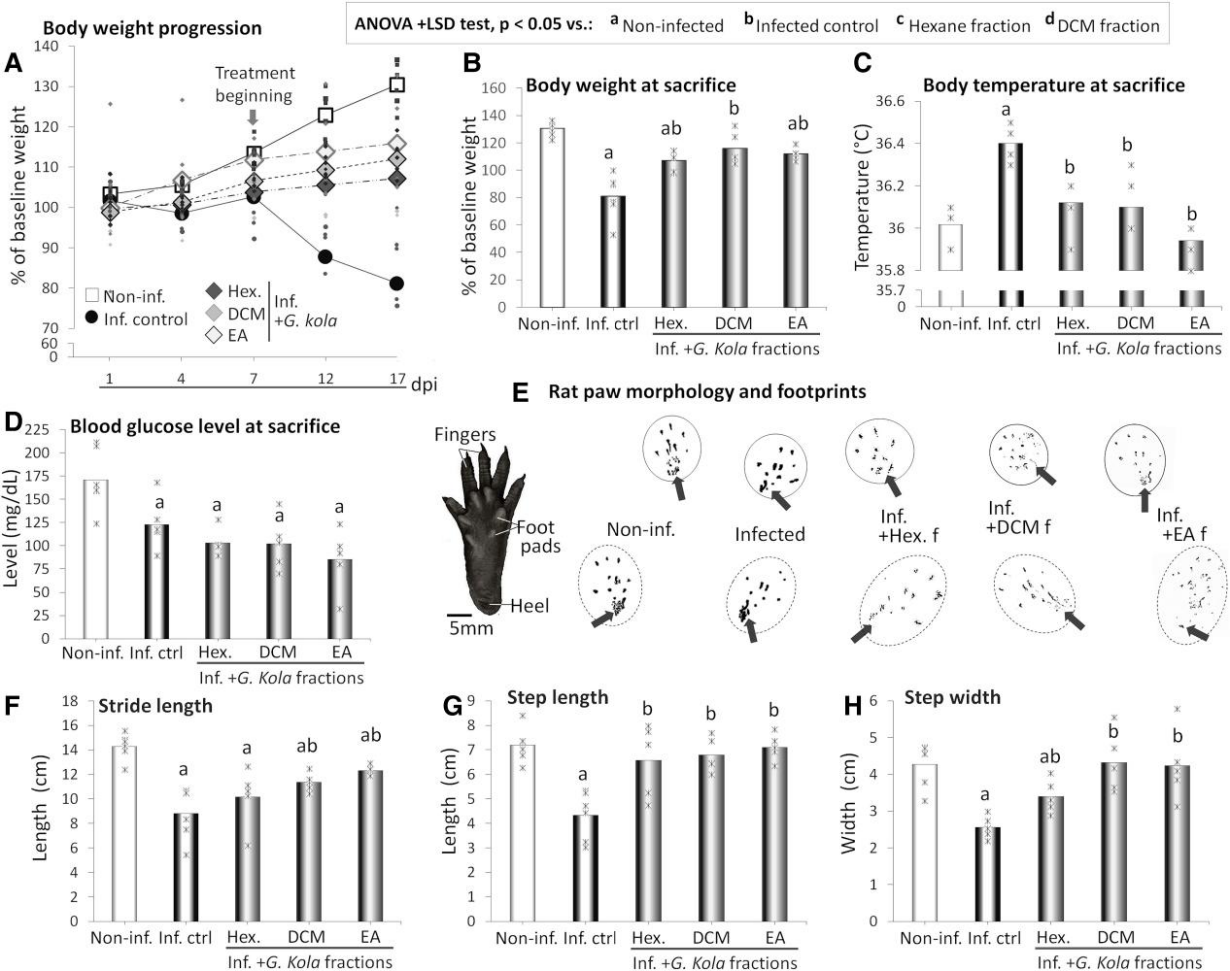
**Physiological parameters and gait quality indicators**. (**A–C**) Body weight progression (**A**), average values of body weight (**B**), and body temperature (**C**), and blood glucose (**D**) at sacrifice of infected animals treated with *G. kola* hexane fraction (Hex. f.), dichloromethane fraction (DCM f.) and ethyl acetate fraction (EA f.). (**E–H**) Footprints of representative animals of these groups (**E**) and stride length (**F**), step length (**G**) and step width (**H**) of the same animals. Note that unlike the infected control, and as the non-infected, the treated animals tend to use both heels and footpads (**E**). *N* = 6 animals/group. Critical value of ANOVA *F*-score: 4.35. Dpi, days post-infection; Inf. Ctrl., infected control animals; Non-inf., non-infected animals.

Figure 1A shows the progression of body weights of infected rats treated with fractions of *G. kola*, while Fig. 1B shows the weights of these animals at sacrifice.

The rats of the non-infected group appeared healthy and displayed a linear increase in body weight throughout the experimentation (*y* = 7.09*x* + 93.75, *R*² = 0.98), while infected control animals ceased to grow in weight around dpi 7 and displayed increasingly high decreases in body weight (Fig. 1A), hence appeared skinny. Instead, this inflection in body weight was prevented in the infected groups treated with fractions of *G. kola*, which showed increases in body weight throughout the experimentation as the non-infected animals, with *y* = 1.98*x* + 97.50, *R*² = 0.98 for hexane fraction, *y* = 3.44*x* + 95.35, *R*² = 0.98 for DCM fraction and *y* = 3.90*x* + 98.01, *R*² = 0.93 for EA fraction (Fig. 1A). All fractions of *G. kola* markedly improved body weight values of the infected animals, with significant increases compared with the infected control group at sacrifice (*P* < 0.001) (Fig. 1B). However, only treatment with the DCM fraction restored the body weight to values comparable with non-infected animals (*P* = 0.088 versus non-infected group) (Fig. 1B).

Treatment with fractions of *G. kola* prevented the increase in body temperature observed at sacrifice in the infected control group (*P* = 0.005, *P* = 0.006 and *P* < 0.001 for hexane, DCM and EA fractions, respectively) (Fig. 1C) and maintained the body temperature to values comparable with non-infected animals (*P* = 0.173, *P* = 0.281 and *P* = 0.287, respectively) (Fig. 1C). Instead, treatment with *G. kola* extracts did not improve the low level of blood glucose observed in untreated LPD-fed infected rats, and EA fraction further decreased it, although with a high inter-individual variability (*P* = 0.005, *P* = 0.017 and *P* = 0.003 for hexane, DCM and EA fractions, respectively) (Fig. 1D).

#### Gait quality indicators

Figures 1E–H show the footprints of representative animals (Fig. 1E) and the stride lengths (Fig. 1F), step lengths (Fig. 1G) and step widths (Fig. 1H) of infected rats treated with fractions of *G. kola*. Unlike the non-infected rats, the infected control animals tended to use their footpads more and their heels less during locomotion, particularly at hindlimbs (Fig. 1E). This sign of gait alteration was mitigated or prevented by treatment with fractions of *G. kola*, in particular DCM and EA fractions, as the infected animals treated used their heels and footpads normally (Fig. 1H). Compared with the non-infected rats, the infected control animals displayed significantly lower stride lengths, step lengths and step widths (*P* < 0.001) (Fig. 1F–H).

Except for the treatment with the hexane fraction that did not improve the stride length (*P* = 0.385 versus infected control group), treatments with fractions of *G. kola* resulted in significantly higher stride length values compared with the infected control group (*P* < 0.001 for both DCM and EA fractions), although these values were still lower than non-infected group values (*P* = 0.009 and *P* < 0.001, respectively) (Fig. 1F). Instead, all groups treated with fractions of *G. kola* showed improvements in step length (*P* = 0.028 for hexane fraction, and *P* < 0.001 for both DCM and EA fractions) (Fig. 1G) and step width (*P* = 0.011 for hexane, *P* = 0.007 for DCM and *P* = 0.017 for EA fractions) (Fig. 1H). Notably, the infected animals treated with DCM and EA fractions, and to a lesser extent hexane fraction, presented with values comparable with the non-infected animals for both step length (*P* = 0.404 for hexane, *P* = 0.313 for DCM and *P* = 0.728 for EA fractions versus non-infected group) (Fig. 1G) and width (*P* = 0.011 for hexane, *P* = 0.992 for DCM and *P* = 0.872 for EA fractions versus non-infected group) (Fig. 1H).

### OFT cognitive and motor indicators

#### Locomotion

**Figure 2:**
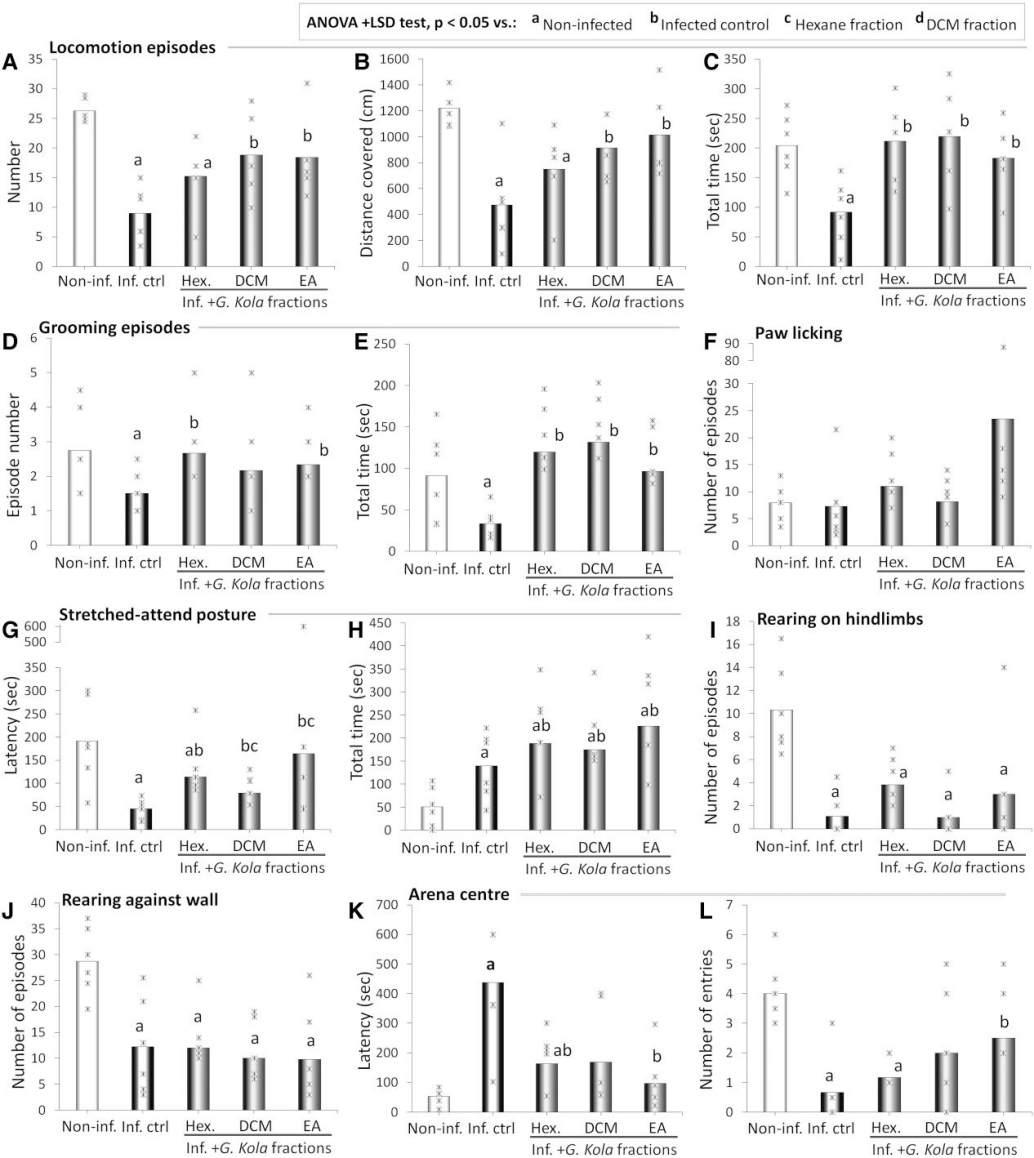
**Changes in OFT cognitive and motor indicators**. Number of locomotion episodes (**A**), distance covered in the arena (**B**), and time spent walking (**C**). Number (**D**) and total time (**E**) of grooming episodes, and number of paws licking episodes (**F**). SAP latency (**G**) and time (**H**), and number of episodes of rearing on hindlimbs (**I**) and against the wall (**J**). Arena centre latency (**K**) and entries (**L**) of infected animals treated with *G. kola* hexane fraction (Hex.), DCM f., and EA f.. Note that overall, the EA f. has the strongest effects. *N* = 6 animals/group. Critical value of ANOVA *F*-score: 4.35. Inf. Ctrl., infected control animals; Non-inf., non-infected animals.

Figures 2A–C present the number of locomotion episodes (Fig. 2A), the distance covered in the arena (Fig. 2B) and the time spent walking (Fig. 2C) of infected rats treated with fractions of *G. kola*. Compared with the non-infected rats, the infected control animals presented with lower locomotion episode numbers, shorter distances covered in the arena and decreased active times (*P* < 0.001) (Fig. 2A–C). Except for the time spent active (*P* = 0.018 versus infected control group and *P* = 0.871 versus non-infected group) (Fig. 2C), these alterations were not corrected in rats treated with the hexane fraction of *G. kola* (*P* = 0.321 versus infected control group and *P* = 0.009 versus non-infected rats for the distance covered in the arena and *P* = 0.200 and 0.031, respectively, for the distance covered) (Fig. 2A and B).

Instead, values comparable with non-infected animals were observed in the infected groups treated with the EA fraction, and to a lesser extent, the DCM fraction: (i) in the locomotion episode number (*P* = 0.036 versus infected control and *P* = 0.091 versus non-infected animals for the DCM fraction and *P* = 0.038 versus infected control and *P* = 0.075 versus non-infected animals for the EA fraction) (Fig. 2A); (ii) in the distance covered (*P* = 0.023 versus infected control and *P* = 0.051 versus non-infected animals for the DCM fraction and *P* = 0.025 versus infected control and *P* = 0.267 versus non-infected animals for the EA fraction) (Fig. 2B); and (iii) in the time spent walking (*P* = 0.033 versus infected control and *P* = 0.758 versus non-infected animals for the DCM fraction and *P* = 0.031 versus infected control and *P* = 0.579 versus non-infected animals for the EA fraction) (Fig. 2C).

#### Grooming

Figures 2D–F present the number (Fig. 2D) and total time (Fig. 2E) of grooming episodes, and the number of paws licking episodes (Fig. 2F) of infected rats treated with fractions of *G. kola*. Compared with the non-infected rats, the infected control animals had significantly lower grooming episode number (*P* = 0.035) (Fig. 2D) and time (*P* = 0.008) (Fig. 2E). Significantly higher values of grooming episode number and time compared with the infected control rats were observed in the infected rats treated with the hexane fraction (*P* = 0.023 and 0.002, respectively), the EA fraction (*P* = 0.026 and 0.004, respectively), but only for the grooming time in the group treated with DCM fraction (*P* = 0.206 and 0.001, respectively) (Fig. 2D and E). Instead, no statistically significant inter-group difference was observed in the paw-licking episode number due to high inter-individual variability, particularly in the EA fraction where a high increase was observed (Fig. 2C).

#### SAPand rearing

Figures 2G–J show the SAP latency (Fig. 2G) and time (Fig. 2H), and the number of episodes of rearing on hindlimbs (Fig. 2I) and against the wall (Fig. 2J) of infected rats treated with fractions of *G. kola*. Compared with the non-infected rats, the infected control rats displayed a significantly lower SAP latency (*P* = 0.017) (Fig. 2G) and a significantly higher SAP time (*P* = 0.003) (Fig. 2H). Compared with the infected control rats, animals treated with all fractions of *G. kola* showed higher SAP latencies (*P* = 0.037, 0.007 and 0.002 for hexane, DCM, and EA fractions, respectively), but unlike the other fractions (*P* = 0.317 and 0.451 versus non-infected group for DCM and EA fractions, respectively), treatment with hexane did not result in values comparable with non-infected animals (*P* = 0.004 versus non-infected group) (Fig. 2G). Intriguingly, the infected groups treated with fractions of *G. kola* displayed marked increases in SAP time compared with both the infected control group (*P* = 0.045 for hexane, *P* = 0.021 for DCM and *P* < 0.001 for EA fractions) and the non-infected group (*P* = 0.001 for hexane, *P* = 0.004 for DCM and *P* < 0.001 for EA fractions) (Fig. 2H). Compared with the non-infected rats, the infected control rats had significantly decreased episode numbers of rearing against the wall (*P* = 0.002) (Fig. 2I) and rearing on hindlimbs (*P* < 0.001) (Fig. 2J). On the other hand, no significant improvement was observed for any of these parameters in the infected groups treated with fractions of *G. kola* (*P* > 0.05 versus infected control group) with significant differences compared with the non-infected group (*P* < 0.001) (Fig. 2I and J).

#### Arena centre entries

Figures 2K and L show the latency to the first entry (Fig. 2K) and the number of entries (Fig. 2L) to the arena centre of infected rats treated with fractions of *G. kola*. Compared with the non-infected rats, the infected control rats had a significantly higher arena centre latency (*P* = 0.002) (Fig. 2K) and lower centre entries (*P* < 0.001) (Fig. 2L). These parameters were improved up to non-infected-like values in the group treated with the EA fraction (*P* = 0.009 and 0.021 versus infected control group and *P* = 0.269 and 0.252 versus non-infected group, respectively for the centre latency and entries), and to a lesser extent in the group treated with the hexane fraction (*P* = 0.037 and 0.021 versus infected control group and *P* = 0.236 and *P* < 0.001 versus non-infected group, respectively for the centre latency and entries) (Fig. 2K and L). High inter-individual variability prevented the statistical significance of the decrease in centre latency (Fig. 2K) and increase in centre entries (Fig. 2L) observed in the infected animals treated with DCM fraction compared with the infected control group.

### EPM cognitive and motor indicators

#### Motor indicators

**Figure 3:**
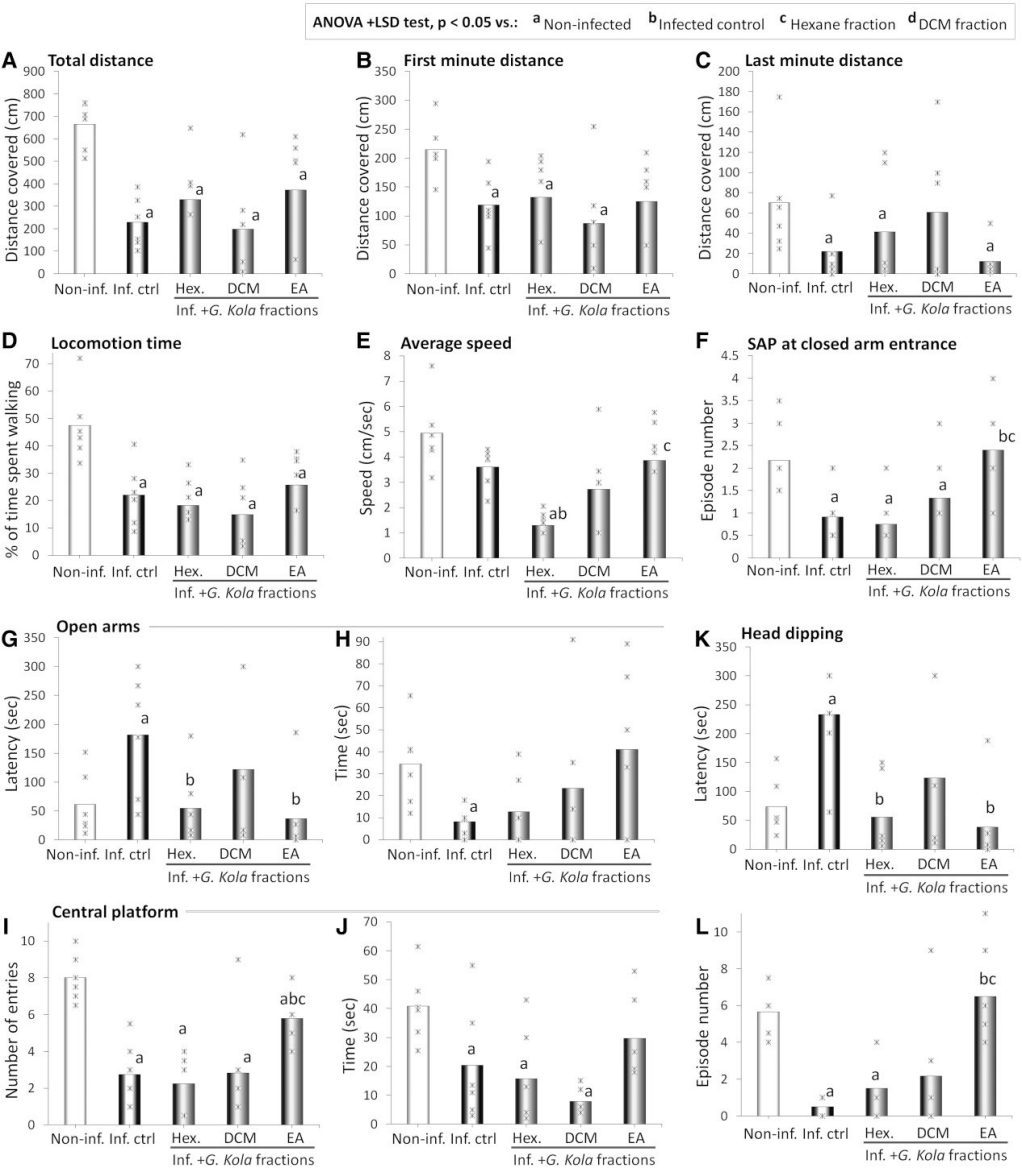
**Changes in EPM cognitive and motor indicators**. Total (**A**), first minute (**B**) and last minute (**C**) distances, time spent walking (**D**), and average speed (**E**); number of episodes of SAP at closed-arm entrance (**F**), open-arm latency (**G**) and time (**H**); central platform entries (**I**) and time (**J**) and head dipping latency (**K**), and episode number (**L**) of infected animals treated with *G. kola* hexane fraction (Hex.), DCM f., and EA f. *N* = 6 animals/group. Critical value of ANOVA *F*-score: 4.35. Inf. Ctrl., infected control animals. Non-inf., non-infected animals.

Figures 3A–E show the distances covered (Fig. 3C), the time spent walking (Fig. 3D) and the speed (Fig. 3E) in the EPM maze of infected rats treated with fractions of *G. kola*. Compared with the non-infected rats, the infected control rats covered a significantly lower distance all over the test (*P* < 0.001) (Fig. 3A), particularly in the first minute of the test (*P* = 0.004) (Fig. 3B), and to a lesser extent, in the last minute (*P* = 0.048) (Fig. 3C). Treatment of infected rats with fractions of *G. kola* at the doses tested did not improve these parameters (*P* > 0.05 versus infected control group and *P* < 0.001, *P* = 0.004 and *P* = 0.048 versus non-infected group for the total distance covered over the test, in the first minute, and in the last minute, respectively) (Fig. 3A–C).

Compared with the non-infected rats, the infected control rats spent significantly less time walking (*P* < 0.001) (Fig. 3D) and displayed a non-significant decrease in the average speed (*P* = 0.082) (Fig. 3E). These parameters were not improved in infected rats by fractions of *G. kola* at the doses tested (Fig. 3D and E). The locomotion times in the groups treated were comparable with the infected control group and significantly lower than non-infected group values (*P* = 0.643, *P* = 0.323 and *P* = 0.667 versus infected control group and *P* = 0.002, 0.004, and 0.028 versus the non-infected group for hexane, DCM and EA, respectively) (Fig. 3D). Instead, while treatment with hexane further decreased the average speed (*P* < 0.001 versus both infected control and non-infected groups), high inter-individual variability prevented the statistical significance of the increase observed following treatment with EA fraction (*P* = 0.882 versus infected control group and *P* = 0.323 versus the non-infected group), and DCM treatment did not elicit marked changes compared with the infected control group (Fig. 3E).

#### Cognitive indicators

Figures 3F–L show the number of SAP episodes at closed-arm entrance (Fig. 3F), the open-arm latency (Fig. 3G) and time (Fig. 3H), and central platform entries (Fig. 3I) and time (Fig. 3J), as well as the head dipping latency (Fig. 3K) and episode number (Fig. 3L) in the EPM maze of infected rats treated with fractions of *G. kola*. Compared with the non-infected rats, the untreated infected rats fed with LPD had significantly lower SAP numbers (*P* = 0.009) (Fig. 3F), open-arm times (*P* = 0.029) (Fig. 3H), central platform entries (*P* < 0.001) (Fig. 3I) and times (*P* = 0.037) (Fig. 3J), and head dipping episode numbers (*P* < 0.001) (Fig. 3L). Instead, they had higher open arm (*P* = 0.019) (Fig. 3G) and head dipping (*P* < 0.001) (Fig. 3K) latencies. Except for open-arm latency that was improved by hexane treatment (*P* = 0.023) (Fig. 3G), treatment of infected rats with hexane fraction did not improve these parameters significantly (*P* > 0.05 versus infected control group and *P* < 0.05 versus non-infected group) (Fig. 3F–L). Treatment of infected rats with DCM fraction improved these parameters but statistical significance was prevented by a high inter-individual variability (*P* > 0.05 versus infected control group and in most cases, *P* > 0.05 versus non-infected group) (Fig. 3F–L).

Instead, treatment of infected rats with the EA fraction resulted in marked improvements in most of these parameters, with statistically significance reaching values comparable, including: (i) in the number of episodes of SAP at closed-arm entrance (*P* = 0.042 versus infected control group and *P* = 0.699 versus non-infected group) (Fig. 3F); (ii) in the open-arm latency (*P* = 0.011 versus infected control group and *P* = 0.541 versus non-infected group) (Fig. 3G); (iii) in the central platform entries (*P* = 0.003 versus infected control group and *P* = 0.030 versus non-infected group) (Fig. 3I) and (iv) in the head dipping latency (*P* < 0.001 versus infected control group and *P* = 0.373 versus non-infected group) (Fig. 3K) and episode number (*P* = 0.015 versus infected control group and *P* = 0.672 versus non-infected group) (Fig. 3L).

Some parameters were improved in infected animals treated with the EA fraction, but without statistical significance, due to high inter-individual variability, namely the open-arm time (*P* = 0.077 versus infected control group and *P* = 0.726 versus non-infected group) (Fig. 3H) and the central platform time (*P* = 0.569 versus infected control group and *P* = 0.366 versus non-infected group) (Fig. 3J).

### Parasite and cell count in the blood

**Figure 4:**
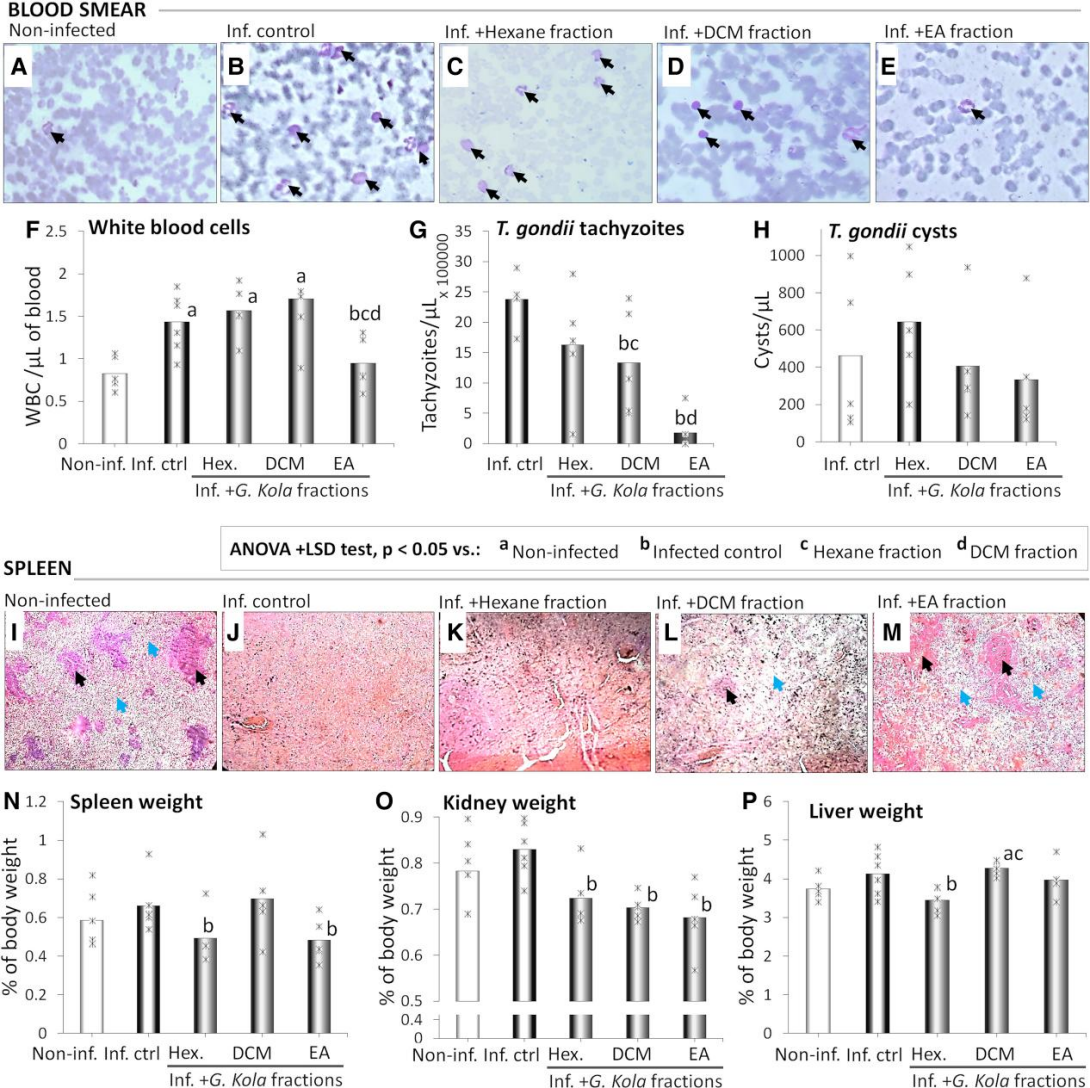
**Changes in blood cells and in organs**. Giemsa-stained blood smears of representative animals treated with *G. kola* hexane (Hex), DCM, and EA fractions (**A–E**). Blood counts of WBC (**F**), *T. gondii* tachyzoites (**G**) and cysts (**H**). H&E-stained spleen sections (**I–M**) and weights of the spleen (**N**), kidney (**O**), and liver (***P***) of the same animals. Black arrows: WBCs. Black arrow heads: spleen white pulp. Blue arrow heads: red pulp. Note the marked inflammation (**B**) and structure loss (**J**) in the infected control group and the effect of the EA f. (**E**). *N* = 6 animals/group. Critical value of ANOVA *F*-score: 4.35. Inf. Ctrl., infected control animals. Non-inf., non-infected animals.

Figures 4A–H show micrographs of the blood smears of representative non-infected (Fig. 4A), infected control (Fig. 4B) and infected animals treated with hexane (Fig. 4C), DCM (Fig. 4D) and EA (Fig. 4E) fractions of *G. kola*. Blood counts of WBCs (Fig. 4F), *T. gondii* tachyzoites (Fig. 4G) and cysts (Fig. 4H) of these animals are also presented. While non-infected animals presented with normal blood smears (Fig. 4A), markedly increased densities of WBCs (Fig. 4B) were observed in blood samples of infected control animals, together with *T. gondii* tachyzoites and cysts (data not shown). Treatment with fractions of *G. kola* induced decreases in observed densities of tachyzoites and WBCs (Fig. 4C–E), with the strongest effects in the group treated with the EA fraction (Fig. 4E).

These observations were confirmed by cell and parasite counts, with significant decreases in WBCs (*P* = 0.038 versus infected control group and *P* = 0.420 versus non-infected group) (Fig. 4F) and *T. gondii* tachyzoites (*P* = 0.045 versus infected control group) (Fig. 4G). Although decreases in infected animals treated with DCM and EA fractions were observed, treatment of infected rats with fractions of *G. kola* at the doses tested did not improve the number of circulating cysts due to high inter-individual variability (*P* = 0.662 and 0.862 versus infected control group for DCM and EA fractions, respectively) (Fig. 4H).

### Organ weight and histology

Figures 4I–P show micrographs of the H&E-stained spleen sections of representative non-infected (Fig. 4I), infected control (Fig. 4J) and infected animals treated with hexane (Fig. 4K), DCM (Fig. 4L) and EA (Fig. 4M) fractions; and weights of the spleens (Fig. 4N), kidneys (Fig. 4O) and livers (Fig. 4P) of the same animals. As illustrated with representative animals, unlike the non-infected rats (Fig. 4I) and the infected animals treated with the EA fraction (Fig. 4M) which presented with normal spleen tissue structures, the infected control rats (Fig. 4J) and the infected rats treated with hexane (Fig. 4K) or DCM (Fig. 4L) fractions lost their spleen tissue structures, with not clearly differentiated white pulp and also had blood vessel leakage. Overall, histopathological analyses of sections of the brain, heart, lungs, kidneys, liver and spleen revealed the presence of blood vessel leakage, mild-to-severe morphological alterations and a few *T. gondii* tissue cysts in animals of the infected control group, which were not observed in the infected animals treated with the EA fraction of *G. kola* (data not shown).

In addition, while no significant inter-group difference was observed in the brain, heart and lung weights (data not shown), non-significant increases were observed in the spleen (Fig. 4N), kidney (Fig. 4O) and liver (Fig. 4P) weights of the infected control group compared with the non-infected group. Despite high inter-individual variabilities at the doses tested, treatments of infected animals with all fractions of *G. kola* decreased the weights of organs to values comparable with the non-infected group, including for the spleen (*P* = 0.040, 0.863 and 0.020 versus infected control group and *P* = 0.372, 0.275, 0.267 versus non-infected group for the groups treated with hexane, DCM and EA fractions, respectively) (Fig. 4N), the kidney (*P* = 0.025, 0.002 and 0.008 versus infected control group and *P* = 0.274, 0.084, 0.081 versus non-infected group for the groups treated with hexane, DCM and EA fractions, respectively) (Fig. 4O), and the liver (*P* = 0.011, 0.463 and 0.569 versus infected control group and *P* = 0.187, 0.008, 0.400 versus non-infected group for the groups treated with hexane, DCM and EA fractions, respectively) (Fig. 4P).

### Density and size of brain neuron nuclei

**Figure 5:**
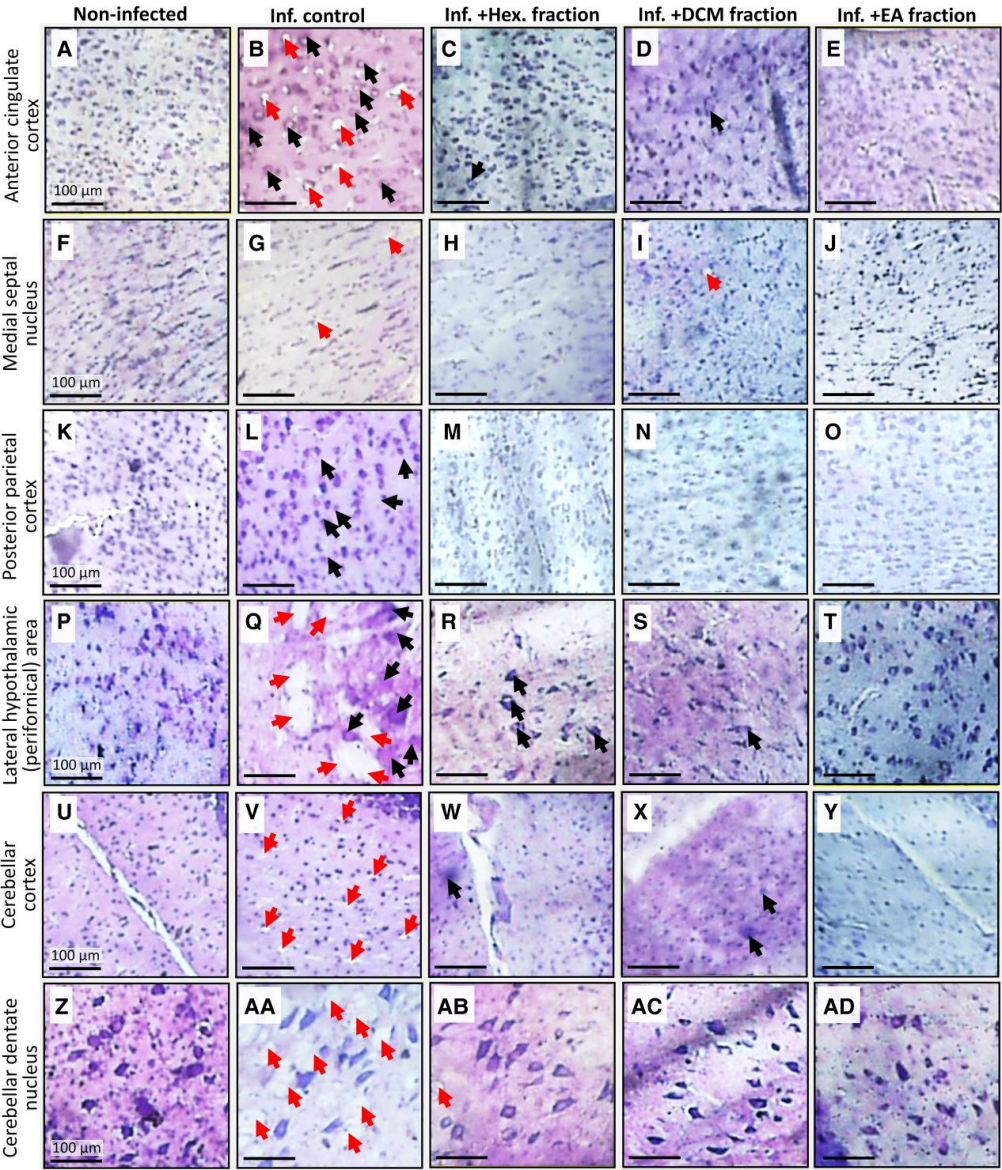
**Brain section micrographs**. Micrographs of cresyl violet-stained brain anterior areas (**A–J**), posterior areas (**K–T**) and cerebellar areas (**U–AD**) of representative infected animals treated with *G. kola* hexane (Hex.), DCM, and EA fractions. Black arrows: enlarged neurons. Red arrows: dead neuron nuclei. Note the neuronal losses and increases in neuron nuclei size in the infected control group and their mitigation by treatments with fractions of *G. kola*.

**Figure 6:**
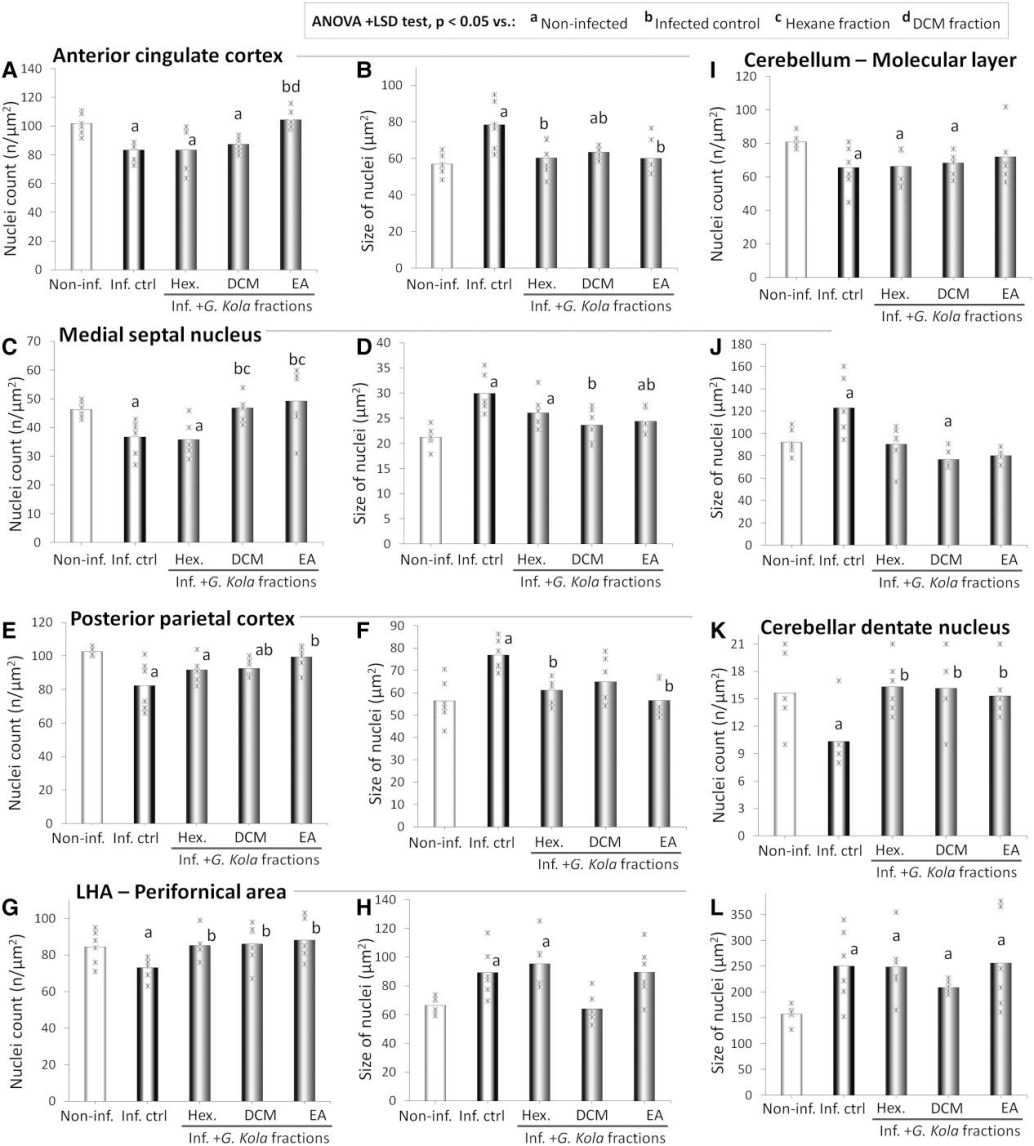
**Brain neuron counts and size**. Density and size of neuron nuclei in anterior (**A–D**) and posterior (**E–H**) brain areas, and in the cerebellar molecular layer (**I, J**) and dentate nucleus (**K, L**) of representative infected animals treated with *G. kola* hexane (Hex.), DCM and EA fractions. Note the neuronal losses and increases in neuron nuclei size in the infected control group (Inf. Ctrl) and their mitigation by *G. kola* treatment. *N* = 6 animals/group. Critical value of ANOVA *F*-score: 4.35. Non-inf., non-infected animals. LHA, lateral hypothalamic area.

Figure 5 shows micrographs of brain sections of representative non-infected, infected control and infected animals treated with fractions of *G. kola* stained with cresyl violet to show neuron nuclei, and Fig. 6 shows the result of neuron nuclei counting and size determination in these animals. The observation of the stained brain sections of infected control animals revealed the following, from anterior to posterior brain areas: (i) neuronal loss and decreased neuronal densities in the anterior cingulate cortex compared with non-infected animals (Fig. 5A and B); (ii) a decreased neuronal density in the medial septal nucleus (Fig. 5F and G); (iii) markedly enlarged neuron nuclei and decreased neuronal density in the posterior parietal cortex (Fig. 5K and L); (iv) highly enlarged neuronal nuclei and massive loss of neurons in the lateral hypothalamic area (Fig. 5P and Q); (v) a decrease in neuronal density in the molecular layer of the cerebellar cortex (Fig. 5U and V) and (vi) a marked decrease in large neuron nuclei density in the cerebellar dentate nucleus (Fig. 5Z and AA). These alterations were prevented or mitigated in infected animals by all tested fractions of *G. kola* (Fig. 5C and AD).

The results of the observations of the cresyl violet-stained sections were confirmed by the counts of neuron nuclei (Table 1) and the estimation of their size (Table 1), as compared with the non-infected group, the infected control rats had significantly lower neuronal densities in the brain areas assessed (*P* < 0.001) (Fig. 6). Infected animals treated with the EA fraction (respectively, hexane and DCM fractions) displayed almost normal neuronal densities (respectively, mild-to-moderate protections of neuronal populations) (Table 1) in the anterior cingulate cortex (Fig. 6A), the medial septal nucleus (Fig. 6C), the posterior parietal cortex (Fig. 6E), the lateral hypothalamic area (Fig. 6G), the molecular layer of the cerebellar cortex (Fig. 6I) and the dentate nucleus (Fig. 6K).

**Table 1:** *P*-values of neuron nuclei count and size inter-group comparisons

	Inf. control	Hexane fraction	DCM fraction	EA fraction
*Nuclei count*				
Anterior cingulate cortex				
Non-infected group	3.0E-5***	0.001**	0.016*	0.773
Infected control group		0.992	0.370	0.013*
Medial septal nucleus				
Non-infected group	0.002**	0.005**	0.880	0.579
Infected control group		0.783	0.005**	0.012*
Posterior parietal cortex				
Non-infected group	4.0E-6***	0.001**	0.001**	0.189
Infected control group		0.106	0.138	0.018*
Lateral hypothalamic area				
Non-infected group	0.028*	0.964	0.894	0.769
Infected control group		0.011*	0.051	0.011*
Cerebellar molecular layer				
Non-infected group	0.007**	0.003**	0.025*	0.234
Infected control group		0.923	0.742	0.635
Cerebellar dentate nucleus				
Non-infected group	0.002**	0.765	0.890	0.892
Infected control group		8.4E-5***	0.003**	0.013*
*Nuclei size*				
** **Anterior cingulate cortex				
Non-infected group	1.2E-6***	0.262	0.020*	0.422
Infected control group		0.002**	0.016*	0.031*
Medial septal nucleus				
Non-infected group	3.9E-6***	0.002**	0.104	0.035*
Infected control group		0.069	0.018*	0.039*
Posterior parietal cortex				
Non-infected group	0.001**	0.217	0.067	0.951
Infected control group		0.034*	0.199	0.031*
Lateral hypothalamic area				
Non-infected group	0.020*	0.011*	0.790	0.064
Infected control group		0.637	0.089	0.987
Cerebellar molecular layer				
Non-infected group	0.037*	0.795	0.045*	0.290
Infected control group		0.095	0.064	0.252
Cerebellar dentate nucleus				
Non-infected group	4.5E-4***	2.2E-6***	0.002**	0.001**
Infected control group		0.978	0.285	0.915

ANOVA + LSD test: *P < 0.05; **P < 0.01 and ***P < 0.001.

Treatment of infected rats with the EA fraction, and to a lesser extent DCM fraction, prevented or mitigated the increase in neuron nuclei size (as indicated by *P* < 0.05 versus infected control group) and maintained nuclei size close to normal values (as indicated by *P* > 0.05 versus non-infected group) in the anterior cingulate cortex (Fig. 6B) and the posterior parietal cortex (Fig. 6F), and less markedly in the medial septal nucleus (Fig. 6C, Table 1). However, fractions of *G. kola*, in particular, the EA fraction, did not prevent neuron nuclei enlargement in the lateral hypothalamic area (Fig. 6H), and the cerebellar molecular layer (Fig. 6J) and dentate nucleus (Fig. 6L), partly due to high inter-individual variabilities for the first two areas (*P* > 0.05 versus both infected control and non-infected groups) (Table 1).

### Plant extraction and phytochemical screening

The extraction yields were 5.9% for the hexane fraction, 5.2% for the DCM fraction and 6.2% for the EA fraction. The secondary metabolites present in the fractions of *G. kola* tested are presented in Table 2. The estimation of concentrations of some secondary metabolites revealed the highest concentrations of phenolic compounds (920.88 ± 3.52 mg of gallic acid equivalents per gram of dry extract against 232.64 ± 4.70 and 176.76 ± 9.41 mg EGAg for DCM and hexane fractions, respectively) and tannins (23.76 ± 2.4 milligrams of tannic acid per gram of dry extract against 18.58 ± 0.23 and 15.03 ± 0.45 mg ETAg for hexane and DCM fractions, respectively) in the EA fraction. The hexane fraction had the highest concentrations of flavonoids that were more concentrated (257.5 ± 5 milligram per equivalent per gram against 215.83 ± 1.67 and 141.38 ± 10.18 mg Eqg for EA and DCM fractions, respectively) and flavanols (74.74 ± 0.30 mg Eqg against 58.57 ± 0.49 and 51.96 ± 0.42 mg Eqg for DCM and EA fractions, respectively).

**Table 2:** Secondary metabolites present in the fractions of *G. kola*.

	Hexane fraction	DCM fraction	EA fraction
Alkaloids	+	+	+
Anthocyanins	−	−	−
Catechic tannins	−	−	+
Coumarins	+	+	−
Flavonoids	+	+	−
Gallic tannins	−	−	−
Mucilage	−	−	−
Phenolic groups	+	+	+
Quinones	−	+	+
Saponins	+	+	−
Steroids	−	−	−
Terpenoids	+	+	+

+ indicates presence; − indicates absence.

## Discussion

The results of this study support that treatment of *T. gondii*-infected rats with fractions of the methanolic extract of *G. kola* seeds, particularly the EA fraction, improved the animal condition and cognitive and motor functions, in part by reducing the parasite load and inflammation at the systemic level and by preventing the hypertrophy of neuronal nuclei and neuronal loss.

The infected control animals presented with typical signs of *T. gondii*-induced systemic disease, i.e. a severe cachexia and a sustained fever^51,52^ probably exacerbated in this model by LPD, hence undernutrition in proteins induced mild-to-moderate immune dysfunctions.^21,22^ These signs included the animal appearance, markedly lower body weights, and higher body temperatures at sacrifice compared with non-infected animals. Also supporting the occurrence of the detrimental inflammatory processes driving the systemic disease associated with active *T. gondii* infection: (i) the infected control animals displayed increases in weights of detoxifying organs, including the liver,^53,54^ kidney^55,56^ and spleen^57,58^; (ii) structure loss was observed in the spleen with the disappearance of the white pulp, possibly indicating functional asplenia or spleen hypofunction that may have resulted from systemic disease^59,60^ and (iii) large numbers of tachyzoites were observed in the blood, together with marked increases in circulating WBCs, which is typical of the first stage of active toxoplasmosis experimental in immunocompetent intermediate hosts.^61,62^

Overall, the infected animals treated with the EA fraction of *G. kola*, and to a lesser extent DCM fraction, did not show most of these signs of *T. gondii*-induced systemic disease, and had values comparable with non-infected animals for body temperature, organ weights and spleen structure. They also showed a significant body weight increase compared with the infected control animals, suggesting improvement of the animal condition, and decreases in tachyzoite number, indicating antiparasitic activities. This could have been expected considering that EA extracts of *G. kola* seeds were reported to have antiparasitic activities against other apicomplexans, in particular *Plasmodium* species,^63-65^ and also many antiplasmodial agents have anti-*Toxoplasma* activities, including lumefantrine^66^ and artemisinin derivatives such as artemether, artesunate and dihydroartemisinin.^67,68^ These observations suggest that treatment with *G. kola* extracts, and particularly, the EA fraction, mitigated the progression of *T. gondii*-induced systemic disease, possibly via the reduction of the parasite load in the blood and the systemic inflammation. However, fractions of *G. kola* did not improve the blood glucose levels of infected animals, and the EA fraction seemed to further decrease it, although with a high inter-individual variability. Although it may not be a sign of toxicity-induced hypoglycaemia but instead the product of the well-established potent hypoglycaemic activities of the EA fraction of *G. kola*,^26,28,29^ this hints at a problem that should be considered in future and efforts should be aimed at developing improved traditional medicines from the seed extracts of *G. kola*.

In addition, numerous signs of motor and cognitive impairments were observed in the infected control rats, but not in the infected animals treated with the EA fraction of *G. kola* seeds, compared with the non-infected animals. Notably, they included significant: (i) decreases in the total distance covered and in the time spent exploring the OFT arena and EPM maze novel environments, which may result from asthenia associated with the systemic disease^51,69^ and/or anxiety^70,71^; (ii) decreases in the number of visits to the OFT arena central area and EPM open arms, which are also major indicators of anxiety^72,73^; (iii) reductions in grooming episode number and time, also suggesting the occurrence of mood disorders, in particular depression^74,75^ and (iv) decreases in stride length, step length and step width, which may indicate neurologic deficits.^76^ Moreover, cresyl violet-stained brain section observation, neuron counting and neuron nuclei size determination revealed neuronal loss in all the brain areas critical for cognitive and motor responses examined in infected control animals, with the enlargement of neuron nuclei in some of these areas. The areas examined included the following: (i) the anterior cingulate cortex that is involved in action, emotion and memory, with a cardinal role in the control the expression of contextual fear generalization^41,42^; (ii) the medial septal nucleus that is critical for learning and memory and which prevented sepsis-induced cognitive deficits in mice^43,44^; (iii) the posterior parietal cortex, which is an associative region comprising the primary somatosensory areas^45^; (iv) the perifornical zone of the lateral hypothalamic area, which is critical for various physiological functions, including the promotion and stabilization of active-arousal and drive to eating^46,47^; (v) the cerebellar molecular layer which contains inter-neurons that are key elements of cerebellar network computation and behaviour^48^ and (vi) the cerebellar dentate nucleus whose neurons are commonly lost in various neurodegenerative disorders with cerebellar signs.^49,50^ Altogether, these observations suggest that infected control animals displayed major signs of brain functional alterations, including cognitive and motor impairment, emerging partly from neuronal loss. Neurologic deficits and psychiatric signs were expected in animals with untreated severe toxoplasmosis.^61,62^ However, enlargements of nuclei and of entire neuronal cells are a typical feature of various neurodegenerative disorders, where they would emerge as an attempt to compensate for loss of function resulting from neuronal loss. Notably, accounting in the pathophysiology of these diseases, cell enlargement increases the risk of neuron early death.^77-79^ In addition, and potential topics for future studies, recently reported detrimental changes in gut microbiota structure^80,81^ and organ-to-brain signalling^58,82^ probably accounted among the mechanisms of extensive neuronal loss observed in this study.

Interestingly, in this study, treatment with the EA fraction and, to a lesser extent, the DCM fraction, prevented or delayed the development of almost all cognitive and motor impairment signs in *T. gondii-*infected animals, and prevented neuronal loss in most of the areas examined. Thus, these *G. kola* extracts have a promising therapeutic potential against cerebral toxoplasmosis. These effects emerged in part from the well-established neuroprotective properties of the EA extract of *G. kola* seeds.^26,27,32^ Furthermore, the phytochemical screening of the EA fraction of *G. kola* revealed that it had a comparable secondary metabolite profile as EA extracts previously reported neuroprotective, anti-inflammatory, antiparasitic and antioxidative activities.^25-29^ Hence, future studies performing more advanced fractionation of the EA fraction of *G. kola* such as gas chromatography-mass spectrometry or high-performance liquid chromatography-mass spectrometry chromatography may reveal compounds with strong therapeutic potentials against both *T. gondii*-associated systemic disease and cerebral toxoplasmosis.

## Conclusion

The tested fractions of the methanolic extract of *G. kola* seeds showed positive activities against undernutrition-potentiated *T. gondii*-associated systemic disease and cerebral toxoplasmosis in infected rats exclusively fed with a LPD. It is revealed by the prevention or mitigation of signs of systemic disease and cognitive and motor impairment, such as marked decreases in body weight, increases in body temperature, signs of anxiety, depression and neurologic deficits, as well as neuron enlargement and loss. The EA fraction of *G. kola* seeds presented the most beneficial effects and almost totally reversed the alterations observed in the infected control animals, suggesting a good therapeutic potential against cerebral toxoplasmosis. Future studies using more advanced fractionation techniques may reveal the active principles accounting for the activities of fractions of *G. kola* against *T. gondii*-associated systemic disease and cerebral toxoplasmosis.

## Data Availability

Data will be made available upon reasonable request.
